# A preclinical evaluation of polypropylene/polylacticacid hybrid meshes for fascial defect repair using a rat abdominal hernia model

**DOI:** 10.1371/journal.pone.0179246

**Published:** 2017-06-09

**Authors:** Daniela Ulrich, Isabelle Le Teuff, Stephanie Huberlant, Patrick Carteron, Vincent Letouzey, Renaud de Tayrac

**Affiliations:** 1 Medical University Nimes, 4 Rue du Professeur Robert Debré, Nîmes, France; 2 Medical University Graz, Auenbruggerplatz 14, Graz, Austria; 3 Aspide Medical, 246 Allée Lavoisier, La Talaudiere, France; Kyoto Daigaku, JAPAN

## Abstract

**Objectives:**

Synthetic mesh surgery for both abdominal and urogenital hernia repair is often unsatisfactory in the long-term due to postoperative complications. We hypothesized that a semi-degradable mesh hybrid may provide more appropriate biocompatibility with comparable mechanical properties. The aim was to compare its in vivo biocompatibility with a commercial polypropylene (PP) mesh.

**Methods:**

72 rats were randomly allocated to either our new composite mesh (monofilament PP mesh knitted with polylactic-acid-fibers (PLA)) or to a commercially available PP mesh that was used as a control. 15, 90, and 180 days after implantation into the rat abdomen mesh tissue complexes were analysed for erosion, contraction, foreign body reaction, tissue integration and biomechanical properties.

**Results:**

No differences were seen in regard to clinical parameters including erosion, contraction or infection rates between the two groups. Biomechanical properties including breaking load, stiffness and deformation did not show any significant differences between the different materials at any timepoint. Macrophage staining did not reveal any significant differences between the two groups or between timepoints either. In regard to collagen I there was significantly less collagen I in the PP group compared to the PP/ PLA group at day 180. Collagen III did not show any significant differences at any timepoint between the two groups.

**Conclusion:**

A PP/PLA hybrid mesh, leaving a low amount of PP after PLA degradation seems to have comparable biomechanical properties like PP at 180 days due to enhanced collagen production without significant differences in erosion, contraction, herniation or infection rates.

## Introduction

Incisional ventral hernias are common side effects after abdominal surgery and occur between 31 and 49% [1–3]. Similarly, pelvic organ prolapse (POP), a herniation of the female genital organs into the vagina affect between 7 and 23% of women after childbirth [4]. Most common side effects of hernia repair with non-degradable meshes include infection, pain, hematoma, bowel lesions, urological injuries, or severe bleeding with all of those complications being below 1% intra- and perioperatively and the severity of complications varying depending on the procedure performed and on patient characteristics [5, 6]. Native tissue repair has long been the gold standard for hernia repair, however recurrence occurs in up to 17% after abdominal hernia repair [7] and in up to 30% after POP repair [8], respectively. To decrease recurrence rates synthetic materials have been introduced in hernia repair several years ago [3–5]. The recurrence rates for ventral hernia repair with mesh surgery can be decreased to 10% [3] and halfened for POP repair [9]. Exogenic materials provide the necessary support and reinforce weakened tissue while undergoing incorporation at the treatment site [6]. A very large variety of implant materials are currently available for this purpose. The ideal material has been defined as biocompatible, adequately mechanically strong, physical resistant to surrounding tissues, available in a convenient format and affordable for clinical use [7]. Available materials are knitted from monofilament yarn with relatively large pore sizes, in order to allow tissue ingrowth [10]. Poly-propylene (PP) is probably the most widely used synthetic material [9]. This non-degradable material preserves its integrity, and strengthens the fascia both by mechanical tension and by induction of an inflammatory reaction that also causes excessive scar formation leading to local side effects, such as discomfort, pain and erosions in approximately 10% of all patients [5, 6, 11–13].

Previously we evaluated soft-tissue healing after poly (lactic acid) (PLA(94)) mesh implantation in vivo [14] and in a rat model [15]. The advantage of PLA is its biodegradability leaving no foreign tissue *in vivo*. The inflammatory response in PLA(94) was significantly less pronounced and collagen organisation significantly better than in PP. In a subsequent study the meshes were previously gamma-ray sterilised with 25, 75 or 125 kGy to accelerate PLA(94) degradation [16]. The higher the level of gamma-radiation, the higher the incidence of abdominal wall herniation (22.2, 31.3 and 52.6% with 25, 75 and 125 kGy, respectively). Tensile strength was dramatically reduced after gamma-ray-sterilised PLA(94) mesh implantation suggesting that gamma-ray sterilised PLA alone would be too fragile to implant.

The aim of this study was to compare the *in vivo* host tissue response of synthetic hybrid meshes fabricated from PP and PLA without predegradation, with a clinical PP mesh, using a rat abdominal hernia model as a preclinical model for hernia repair surgery.

## Materials and methods

### Meshes

In the course of this experiments the following meshes were used: Xlight mesh® (Aspide medical, La Talaudière, France), a monofilament polypropylene (PP) mesh weighing 27g/m^2^, used as a control mesh. Our new composite mesh (PP/PLA) is a monofilament polypropylene (PP) mesh coated with polylactic acid fibers (PLA); consisting of 63,5% PLA and 36,5% PP.

### Animals

All animal experiments were approved by the local Ethics Committee of the French Ministry of Higher Education and Research (registration number: 02488.01) and were in compliance with regulatory guidelines. The experiments were conducted in the animal laboratory of the faculty of medicine of Montpellier with male wistar albino rats, weighing 250–300 g, aged from 3 to 4 months. Male rats have a stable hormone profile; hence they were preferred to female rats in this study. All efforts were made to minimize animal suffering and to use the minimum number of animals necessary to produce reliable scientific data. The animals were in quarantine for 1 week prior to treatment. They were housed in individual cages at 22°C with a humidity rate of 55% (+/-10%) and free access to food (SAFE®) and tap water. They were examined on a regular basis with all procedures respecting the guide of good practices and animal welfare.

### Surgical procedure

**Fig 1:**
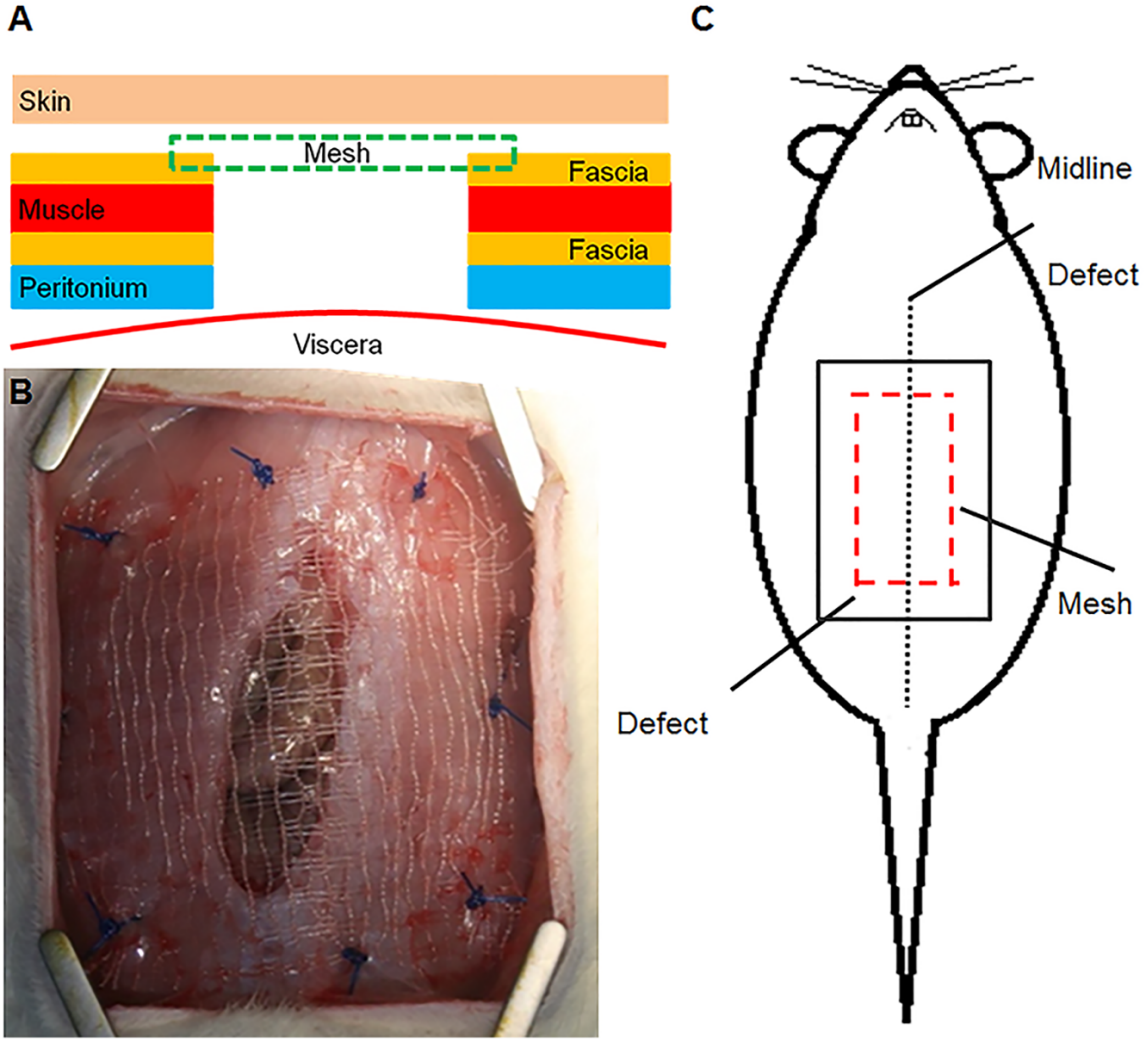
Images of the abdominal hernia model. 1A. Schematic showing the different layers of the abdominal wall and mesh placement. 1B. Photograph of implantation of the PP/PLA mesh. 1C. Schematic of full thickness defect and mesh placement.

Forty-eight animals were randomized to receive one of the two meshes: PP (n = 24) and PP/PLA (n = 24). The rats were anaesthetized by an intraperitoneal injection of ketamine (80mg/kg) and xylazine (5mg/kg). The rats were placed in dorsal position, the abdomen was shaved and prepared with iodine solution and then draped in a sterile fashion. Initially a subcutaneous injection of xylocaine (0,1%) was made to minimize postoperative pain. A ventral 4cm-midline skin incision was made followed by an incision into the abdominal muscle wall consisting of a 15×25mm longitudinal full-thickness defect [17] (Fig 1). Each mesh was spread covering the defect and sutured by 8 stitches of polypropylene 2/0. Before skin closure, a photo was taken from the mesh for further contraction analysis. The skin was closed continuously with polyglactone 3/0. Throughout the study, the animals were visually inspected for signs of dehiscence of the skin wound, seroma formation, wound infection and/or areas of mesh incompatibility.

### Postoperative assessment

Animals were euthanized by intraperitoneal injection of pentobarbital (0,5mL/kg) at 15, 90 and 180 days postoperatively. After euthanization, the abdominal wall was inspected for herniation or erosion by two different observers blinded to the mesh. Erosion was defined as any visible mesh at the outside due to a skin defect; herniation was defined as a visible bulge that could be due to a defect of the mesh itself or due to mesh dehiscence from the abdominal muscle. The skin was retrieved and a photo was taken under the same conditions than during implantation. From the explanted meshes and the host tissue, 2/3 were obtained for biomechanical testing and 1/3 was used for histology and immunohistochemistry.

### Analysis of contraction

All photos were taken with the same camera (Canon EOS 400D digital), at the same resolution (1880X2816 pixels) and at the same distance (40cm) as previously reported [18]. The measurements of the mesh area were done with Image J software, using the blue polypropylene stitches as landmarks.

### Histology

Samples were fixed in 4% formalin at room temperature for 24 to 36h, then moved into 70% ethanol after two washes with PBS to stop fixation and then kept in a refrigerator at 4°C.

The samples were processed according to a HIS 6h program in the Peloris automaton (Leica) for dehydration and then embedded in paraffin to obtain transversal sections through all abdominal wall layers. Paraffin wax blocks were cut into 3–5 mm thick slices and stained with hematoxylin-eosin. One histologist evaluated all tissues and was blinded to the origin of the meshes.

### Immunohistochemistry (IHC)

#### CD68 labeling

Sections underwent dewaxing, rehydrating in graded alcohols followed by antigen retrieval (Roche- Ventana including a protein block). Staining was performed with the CD68 antibody on the Discovery automaton (Roche-Ventana) for macrophage detection (1:200, mouse monoclonal Abcam ab31630) for 1h at room temperature. Detection was performed with a chromoMap DAB detection kit using an OmniMap anti-mouse HRP (Roche-Ventana). Slides were mounted with Pertex mounting medium.

### Immunofluorescence

#### Collagen I and III labeling

For collagen I and collagen III detection, two antibodies were used (Abcam anti-collagen I (ab90395) and anti-collagen III (ab7778)).A tyramide signal amplification kit in order to produce clear and intense fluorescent staining was applied. Slides were mounted with Pertex mounting medium.

### Histomorphometric analysis

#### Image digitization

The IHC slides were digitalised at the Nanozoomer scanner (Hamamatsu) at x20 magnification without Z stack. CD68 slides were digitalised in bright field conditions around the mesh fibres. The labelling percentage on the CD68 stained sections was computed by image analysis from the labelling area divided by the area of the delineated region of interest to be compared between the different samples and groups. Collagen I and collagen III slides were digitalised in fluorescence conditions; the positive area was divided by the total area examined and percentage of collagen content calculated. The collagen I / collagen III ratio was computed from the labelling percentages of each protein.

### Biomechanical testing

All explants were uniaxially tensile tested using an Instron® Tensile Tester (Instron Corp; USA) with a 5 kN load cell. Samples (4x25mm) were tested using a 14 mm gauge length (noted as the initial length) and were pre-loaded to 100 mN at an elongation rate of 10 mm/min to remove any sample slack. Subsequent testing was performed with the longitudinal axis reset to zero elongation as a frame of reference for permanent mesh deformation. Cyclic loading of sub-maximal loads were applied to the mesh using a constant elongation of 50 mm/min and a load from 0.5 N to 5 N. Permanent deformation was assessed as the elongation from the zero point defined after the initial preload. The tissue was then loaded to failure and a load-elongation curve was obtained. The corresponding stress-strain curve was then computed utilizing the cross-sectional area and tissue strain measurements. The parameters describing the mechanical properties of the tissue were then obtained from this curve. The tangent modulus, an indicator of tissue stiffness on a per unit basis, was the maximum slope of the stress-strain curve recorded over a 1% interval of strain. The breaking load was defined as the maximum stress achieved at failure.

### Statistical analysis

Graphpad Prism 7 was used for statistical analysis. Results are reported as median ± SEM for each experimental group (n = 8 animals/ group/ timepoint). Since the data was not normally distributed (D'Agostino & Pearson omnibus normality test), non-parametric analysis using Kruskal–Wallis ANOVA, were undertaken to assess differences between timepoints for the various meshes, followed by Tukey correction. P values < 0.05 were considered as statistically significant.

## Results

4 rats died during the experiment, one for unknown reasons within one week postoperatively in the PP group, 3 died in the 180 day group due to problems with the intestine or massive herniation necessitating euthanasia (1 from the PP group, 2 from the PP/PLA group). All rats showed significant weight gain over time with no significant differences between the two groups (S1 Fig).

### Macroscopic tissue compatibility of new meshes

After euthanization, all animals were inspected for signs of erosion and herniation. Erosion was not evaluated at day 15 because we assumed that skin healing was not finished and that there was an inflammatory response due to suture degradation. In total, in the PP group, the erosion rate was 22%, in the PP/PLA group it was 21%, respectively, with no significant differences between the two groups at any timepoint.

**Fig 2:**
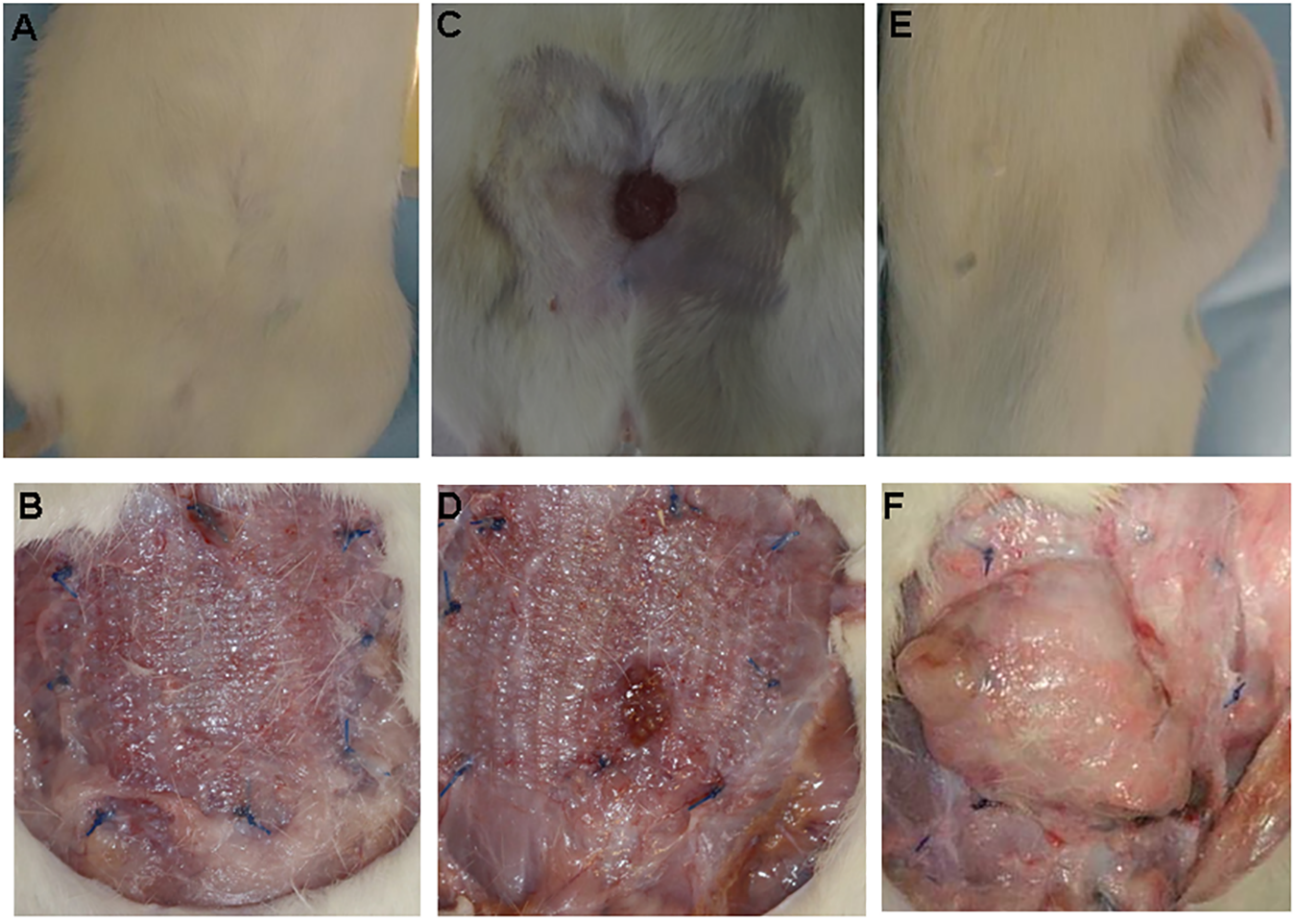
Images taken at euthanization (180 days) frontal view showing normal wound healing of PP mesh with skin (Fig 2A) and without skin (Fig 2B); frontal view showing mesh erosion in a PP mesh (Figs 2C and D). Fig 2 D (sagittal view) and F (frontal view) showing herniation of a PLA mesh.

In regard to herniation rates, no significant differences were observed in the PP (1/7 rats, 14%) and PP/PLA groups (1/ 8 rats, 13%) at 15 days. Similar results were observed at day 90 with no herniation in the PP and one herniation in the PP/PLA group (1/ 8 rats, 13%, p > 0.05). The highest herniation rates were observed at day 180 with 29% in the PP group (2/7 rats) and 33% in the PP/PLA group (2/6 rats, p>0.05). Herniations occurred both in the centre of the mesh (Fig 2F) and along the suturing line (only 2 animals within all groups). Fig 2 shows representative examples of normal wound healing at 180 days (Fig 2A and 2B), erosion (Fig 2C and 2D) and herniation (Fig 2E and 2F).

Contraction was calculated by dividing the postoperative area by the area at time of implantation. The total contraction rate, observed both at 15 and 180 days, was 46 ± 17% in the PP group, 45 ± 15% in the PP/PLA, with no significant differences at any timepoint between any of the groups.

### Biomechanical properties

**Fig 3:**
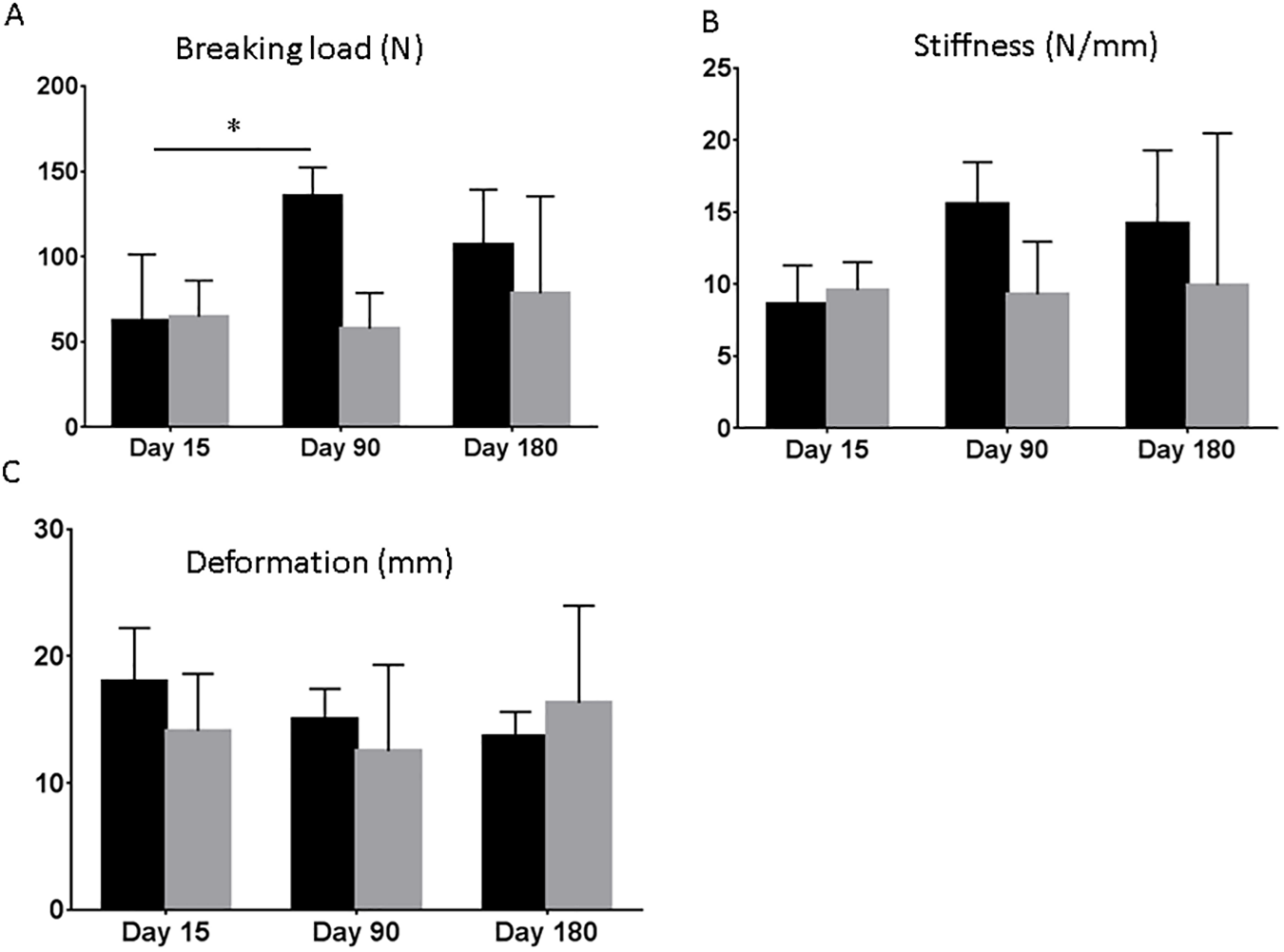
Biomechanical properties in rats implanted with PP and PP/PLA. A. Breaking load after 15, 90 and 180 days. B. Stiffness after 15, 90 and 180 days. C Deformation after 15, 90 and 180 days of implantation. Data are mean ± SD of n = 6 animals/ group *: p< 0.05.

Biomechanical properties including breaking load, stiffness and deformation were evaluated using uniaxial testing. No significant differences were found between the different materials for all parameters at any timepoint, respectively (Fig 3A–3C). There was a trend of lower breaking load and stiffness in the PP/ PLA group at day 90 and 180, however without significant differences (Fig 3A and 3B). Within the PP group, breaking load was significantly higher at day 90 compared to day 15 (p<0.05).

### Inflammatory foreign body reaction

**Fig 4:**
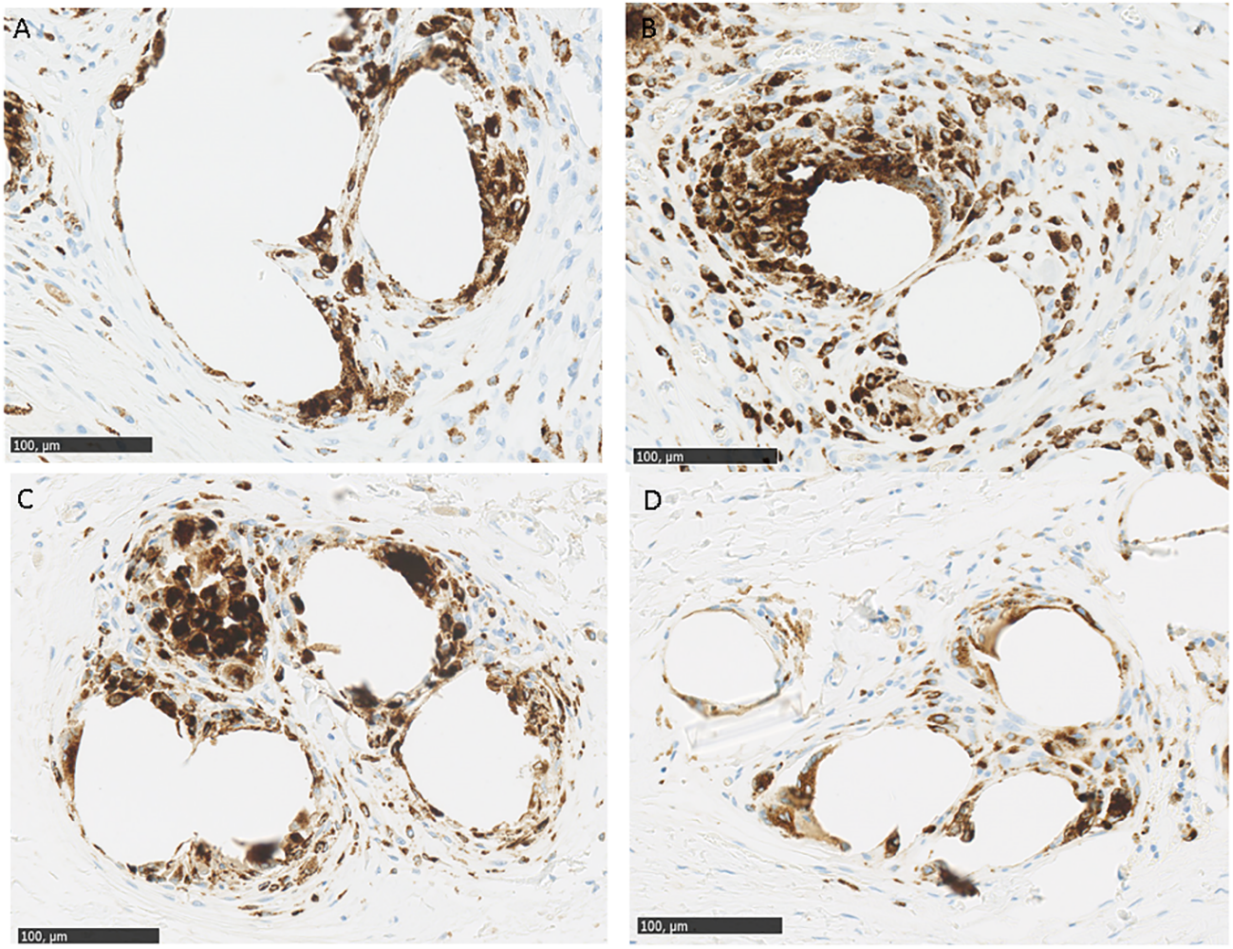
Macrophage staining in explanted meshes. A PP mesh with CD68 staining after 15 days. B. PP/PLA mesh with CD68 staining after 15 days. C. PP mesh with CD68 staining after 180 days. D. PP/PLA mesh with CD68 staining after 15, 90 and 180 days. Scale bars are 100 µm.

**Fig 5:**
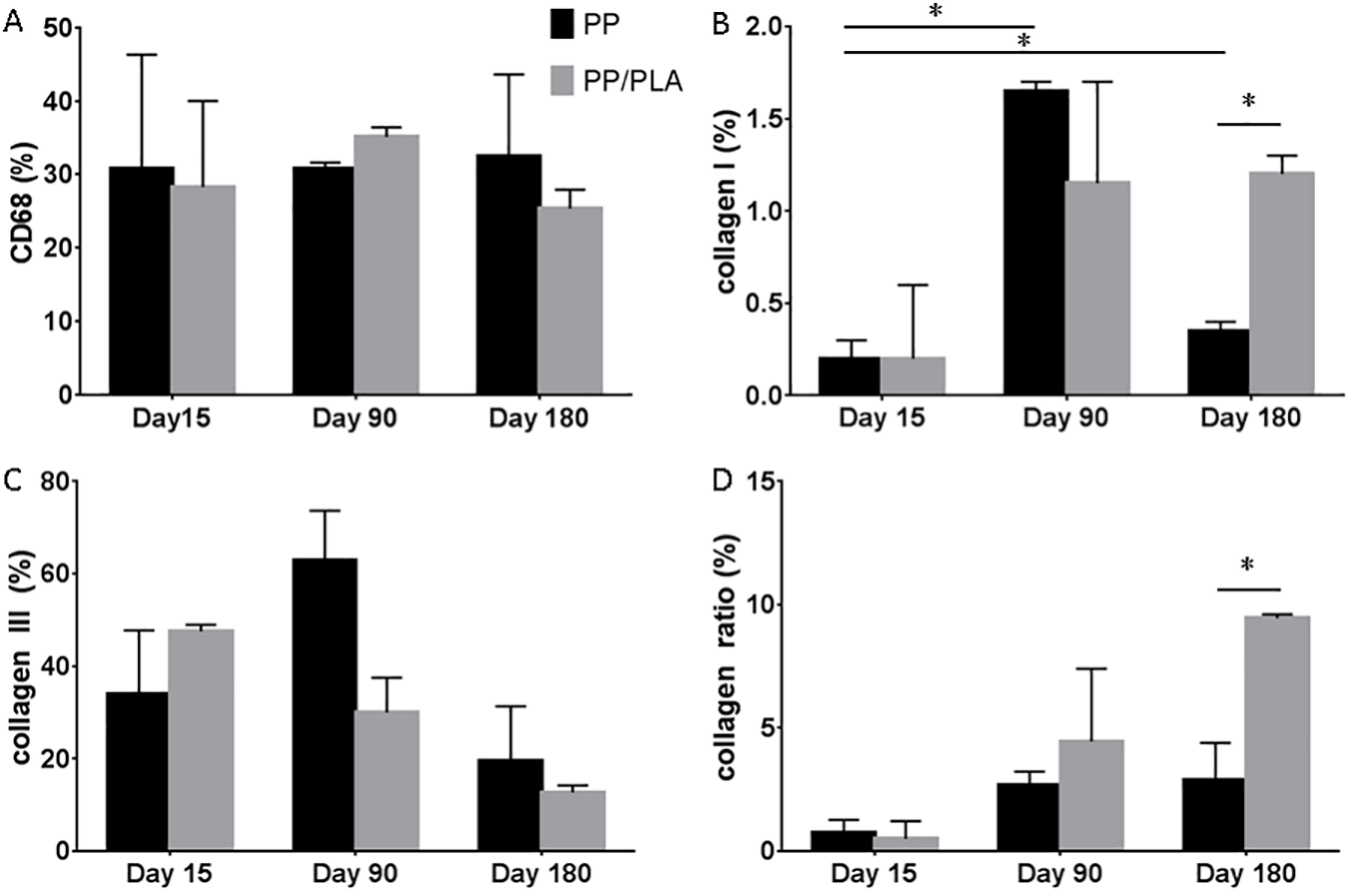
Histological properties in rats implanted with PP and PP/PLA. A. CD68 staining percentage after 15, 90 and 180 days. B. Collagen I staining percentage after 15, 90 and 180 days. C. Collagen III staining percentage after 15, 90 and 180 days. D. Collagen ratio (%) after 15, 90 and 180 days of implantation. Data are mean ± SD of n = 6 animals/ group *: p< 0.05.

Macrophage staining was determined using the CD 68 antibody. Macrophages were present around mesh fibres at all timepoints without any significant differences between any of the groups or between timepoints (Figs 4 and 5). There was only a trend of lower macrophage numbers in the PP/ PLA group at day 180 (Fig 5A).

### Collagen deposition

Collagen deposition was analysed using immunohistochemistry. Collagen I was sparse around the mesh fibres at any timepoint only reaching 2% in the mesh surrounding tissue (Fig 2B). At day 15, there was significantly less collagen I in the PP group compared to day 90 or 180 (p<0.05). Similar trends were observed for the PP/PLA group but without significant differences between the timepoints. At day 180, there was significantly less collagen I in the PP group compared to the PP/ PLA group.

Collagen III was widely spread around mesh fibres with highest levels at day 15 in the PP/PLA group and a decrease over time. There could not be shown any significant differences at any timepoint between the two groups (Fig 2C).

Collagen I/III ratio was lowest at day 15 with increasing values over time reaching a maximum at day 180, especially in the PP/PLA group where the ratio was significantly higher in the PP/PLA group compared to the PP group (p< 0.05).

## Discussion

In this study we investigated biomechanical parameters and histological parameters in a rat abdominal hernia model to assess the *in vivo* biocompatibility of hybrid meshes made from PP and PLA. The results were compared to a clinical PP mesh. Our study shows that the hybrid meshes and the PP meshes behave similarly in terms of biomechanics even though less material remains *in vivo* in the long-term due to degradation of the PLA over time.

All mesh types were generally well tolerated, with significant weight gain in all rats by 180 days and no differences in regard to animal loss or severe complication rates. Herniation and complication rates in the abdominal hernia model differ depending on the surgery technique and material used. Previously herniation rates were reported to be 4% when testing PP meshes [17] and 53% after implantation of irradiated PLA meshes [16]. The herniation rates in this study were between 14 and 33% in a small group of animals with especially the PP/PLA hybrid meshes demonstrating lower herniation rates than expected. The animal loss rate of 7% in this study was slightly higher than previously reported [17, 19], and was mostly because of herniation problems necessitating euthanization according to our ethics protocol.

In line with previous studies examining several commercially available mesh types [20, 21], both our new meshes and the PP meshes showed contraction at all time points. Common causes for pain and tissue erosion are mesh folding and contraction after implantation, hence this should be avoided in human surgery [22].

Biomechanical properties of explanted samples were analysed using uniaxial tensile testing. Biomechanical testing found the hybrid mesh to be weaker at day 90 while this difference could not be seen any more at day 180. This could partially be explained by the lower collagen amount at day 90 in the PP/PLA group and/ or the partial degradation of the PLA. At day 180, the newly formed tissue around mesh fibres seemed to compensate the loss of PLA as can be seen by the comparable strength at 180 days.

A typical inflammatory response was found after implantation with our new hybrid meshes over the time course, largely comparable with the PP meshes. Macrophages are both responsible for the acute inflammation and early vascularisation, but also for the chronic phase. [23].

Collagen I, together with other extracellular matrix proteins influence biomechanical and particularly the viscoelastic properties of tissues. Collagen type III is also widely distributed in soft tissues and can contribute to tissue elasticity. In healing or regenerating tissues an increase in collagen III usually reduces its mechanical strength [24]. Collagen deposition differed clearly between the two groups with more collagen I being laid down at 180 days in the PP/PLA group and lower collagen III levels at the later timepoints suggesting that the hybrid mesh enhances tissue regeneration and that newly laid down collagen might be stronger than that around the PP mesh fibres. The pattern in collagen III deposition is similar to previously reported results [17], with a general decrease of collagen over time.

Strengths of the study are the novelty of the new hybrid mesh combining a non-degradable and a degradable polymer and the long-term follow up. Histological analysis is the most commonly used technique in assessing new meshes [25].

Qualitative and semi-quantitative analysis of tissue by histology/immunohistochemistry has its limitations. Apart from the fact that different parts of the meshes are studied for histology making comparisons difficult [26, 27] more relevant is the problem of subjectivity during the scoring process. In the current study, a software detected the positive staining on each section on the same set of mesh filaments and calculated the percentage stained area. Using the image analysis software excluded any subjective bias and allowed us to precisely focus on the area of interest and to investigate the same area of stained tissue for every image.

This study is not without limitations; one limitation is the fact that we chose the rat abdominal hernia model to assess the in vivo biocompatibility of fascial meshes. The rat probably does not mimic the clinical environment in humans, however, it is frequently used for initial testing for all kind of hernia repair materials including POP [28]. Hence, any interpretation needs to be drawn carefully in animals lacking any pathological conditions. Another limitation is the lack of information regarding intraabdominal adhesion formation since adhesions are an important parameter to measure biocompatibility. Furthermore, based on previous in vitro data the PLA has only partially degraded after 6 months [14]. Longer follow-up studies are necessary to study the biocompatibility of the semidegradable meshes in vivo after complete degradation.

The ideal mesh for hernia repair has not been found yet [22]. In this study we could show that PP/PLA hybrid meshes produce a comparable inflammatory reaction and tissue deposition as do the PP meshes. However, long-term benefits of partially degradable meshes might pose a clear advantage in the clinical setting due to the lower amount of mesh remaining in situ.

In conclusion, the hybrid mesh investigated in this study might be an option for the future treatment of hernia repair.

## Supporting information

S1 Fig Rat weight gain in % at 15, 90 and 180 days after PP or PP/PLA implantation. (TIF)
