# Supplementary figures and images for: A preclinical evaluation of polypropylene/polylacticacid hybrid meshes for fascial defect repair using a rat abdominal hernia model
Source: PLoS One. 2017 Jun 9;12(6):e0179246. doi: 10.1371/journal.pone.0179246 (PMC5466313; doi:10.1371/journal.pone.0179246)

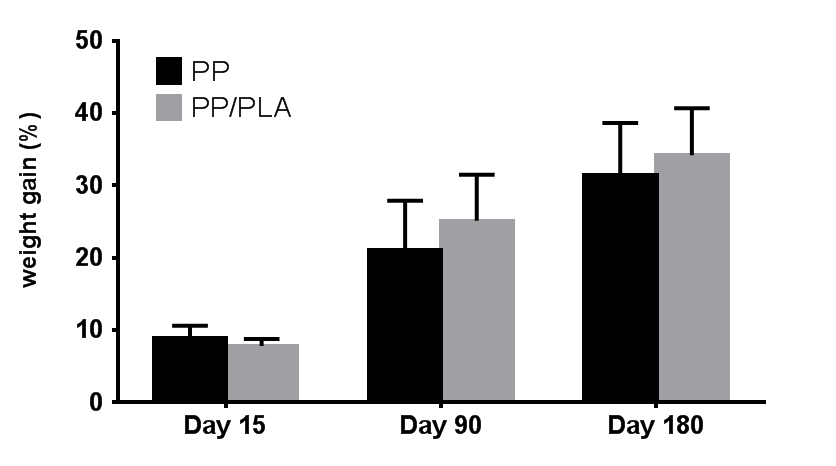

Supplement: S1 Fig — (TIF) [file pone.0179246.s001.tif]
